# Metagenomic next-generation sequencing in the family outbreak of psittacosis: the first reported family outbreak of psittacosis in China under COVID-19

**DOI:** 10.1080/22221751.2021.1948358

**Published:** 2021-07-15

**Authors:** Na Li, Shengjin Li, Wanmei Tan, Hanghang Wang, Huan Xu, Daoxing Wang

**Affiliations:** a Department of Respiratory and Critical Care Medicine, The Second Affiliated Hospital of Chongqing Medical University, Chongqing, People’s Republic of China; b Vision Medicals Center for Infection Diseases, Guangzhou, People’s Republic of China

**Keywords:** Psittacosis, metagenomic next-generation sequencing (mNGS), outbreak, pneumonia, diagnosis

## Abstract

*Chlamydia psittaci* infection in humans, also known as psittacosis, is usually believed to be an uncommon disease which mainly presents as community-acquired pneumonia (CAP). It is usually sporadic, but outbreaks of infection may occasionally occur. In outbreaks, diagnosis and investigations were usually hampered by the non-specificity of laboratory testing methods to identify *C. psittaci*. In this study, we use metagenomic next-generation sequencing (mNGS) in the diagnosis of a family outbreak of psittacosis under COVID-19. Three members of an extended family of 6 persons developed psittacosis with pneumonia and hepatic involvement with common symptoms of fever and weakness. Two newly purchased pet parrots, which had died successively, were probably the primary source of infection. Imagings show lung consolidations and infiltrates, which are difficult to be differentiated from CAP caused by other common pathogens. mNGS rapidly identified the infecting agent as *C. psittaci* within 48 h. The results of this work suggest that there are not characteristic clinical manifestations and imagings of psittacosis pneumonia which can differentiate from CAP caused by other pathogens. The use of mNGS can improve accuracy and reduce the delay in the diagnosis of psittacosis especially during the outbreak, which can shorten the course of the disease control. Family outbreak under COVID-19 may be related to the familial aggregation due to the epidemic. To our knowledge, this is the first reported family outbreak of psittacosis in China, and the first reported psittacosis outbreak identified by the method of mNGS in the world.

## Introduction

Psittacosis is a zoonotic infectious disease caused by the bacterium *Chlamydia psittaci*. Traditionally, psittacines (parrot-type birds) have been considered as the host of the causative bacterium. In addition, many other birds, including wild birds and commercially raised poultry, have also been involved [1]. Pathogens can be transmitted to human beings when the agent is inhaled from bird secretions, dry droppings or dust on feathers. The clinical manifestation of psittacosis in humans may include fever, malaise, myalgia, headache, and chills; cough is characteristically scant or dry and may be absent [2]. Pneumonia, when present, is usually more extensive radiologically than would be expected from clinical signs.

Psittacosis is usually sporadic [3, 4], but outbreaks of infection may occur. The first outbreak was reported by Ritter in 1880 [5], who observed seven cases of unusual pneumonia which happened after getting touch with parrots and finches caged in his brother’s house. Other early outbreaks occurred in Europe and Faroe Islands, which were traced to sick parrots and fulmar petrels [3, 6, 7]. Since Ritter’s original description, there were only some scattered reports of family outbreaks of psittacosis [8–13]. However, there was no report of family outbreak of psittacosis in China until now.

Psittacosis can be diagnosed by serology, isolation of *Chlamydia psittaci*, and molecular detection. Laboratory diagnosis requires meeting any one of the following three criteria: (1) IgM antibody against *C. psittaci* titre detected by MIF of 1:16 or higher; (2) a fourfold or greater increase in antibody titre between serum samples collected 2 weeks apart, using a micro-immunofluorescence (MIF) or complement fixation test (CFT) and (3) isolation of *Chlamydia psittaci* from respiratory secretions [14]. Polymerase chain reaction (PCR) is a more specific and more fast diagnostic test, which is available in specialized diagnostic laboratories [15]. Because of its non-specific symptoms and the limitation of current testing methods, psittacosis is easily underdiagnosed, misdiagnosed [16], and usually treated as atypical pneumonia.

Metagenomic next-generation sequencing (mNGS) is a new tool, which can precisely and rapidly identify potential pathogens, regardless of whether they are bacterial, viral, fungal, or parasitic [17]. Recent studies have highlighted that mNGS is the most promising method for the comprehensive diagnosis of infections, especially for severe pneumonia in intensive-care unit (ICU) settings [18]. This method can objectively and quickly detect more and more rare pathogenic microorganisms in clinical specimens, without the need for specific amplification, and has the advantage for the diagnosis of rare pathogenic microorganisms in difficult cases.

We herein describe the clinical features of a family outbreak of psittacosis pneumonia under COVID-19 which was diagnosed by mNGS after exposure to sick pet parrots, and demonstrate that mNGS is an effective diagnostic method. The family outbreak under COVID-19 may be correlated to the familial aggregation due to the epidemic. To our knowledge, this is the first report of family outbreak of psittacosis identified by mNGS in China.

## Materials and methods

### Study design

We conducted a retrospective case review of 3 members of an extended family of 6 persons admitted successively within 1 week to the Second Affiliated Hospital of Chongqing Medical University, a tertiary teaching hospital in Chongqing, China, with psittacosis pneumonia in December 2020. For each case, data on symptoms, results of laboratory tests, dynamic and comprehensive computed tomography imagings, and clinical course of the disease were extracted from electronic medical records. Additional data on treatments, outcomes, and relevant follow-up data were also collected.

This study was approved by the Ethics Committee of the Second Affiliated Hospital of Chongqing Medical University and conducted in accordance with the Declaration of Helsinki.

### Methods

mNGS was conducted using the following operational steps [19,20]

1. Sample processing and DNA extraction: Clinical samples (sputum or bronchoalveolar lavage fluid, BALF) were collected by following the standards of aseptic processing procedures and treated with enzymes for liquefaction within 24–48 h from collection at 4°C. To selectively enrich the microbial nucleic acid, the samples were processed using the Vision Medicals’ Patho-NET technology to remove host gDNA before DNA extraction. Up to 600 uL samples were transferred to new sterile tubes. Cell wall of microbes was broken by vortex with glass beads following described conditions. DNA was extracted from 300 uL-treated sputum or BALF using the TIANamp Micro DNA Kit (DP316, TIANGEN BIOTECH, Beijing, China) following the manufacturer’s operational manual. The extracted DNA specimens were used for the construction of DNA libraries.

While for clinical sample (blood): Volume of 3–4 mL of blood sample was collected from patients, placed in cell-free blood collecting tubes and stored at room temperature before plasma separation and centrifuged at 1600*g* for 10 min at 4°C. Plasma samples were transferred to new sterile tubes. DNA was extracted from 300 uL of plasma using the TIANamp Micro DNA Kit (DP316, TIANGEN BIOTECH, Beijing, China) following the manufacturer’s operational manual. The extracted DNA specimens were used for the construction of DNA libraries.

(2) Library construction: DNA libraries were constructed through transposase mediated methods (Vision Medicals, China). The quality of the DNA libraries was assessed using a Qsep1 bio-fragment analyzer (BiOptic. Inc., La Canada Flintridge, CA) to measure the adapters and the sizes of fragments before sequencing. The size of qualified library was 300∼500 bp without adapters and PCR dimers, and concentration of the library was greater than 0.5 ng/uL. Finally, qualified DNA libraries were pooled together and sequenced on Nextseq 550 Dx sequencing platform (Illumina, San Diego, CA).
(3) Data Analysis: High-quality sequencing data were generated by removing low-quality, and short (length < 40 bp) reads, followed by computational substractions of human host sequences mapped to the human reference genome (hg38 and YH sequences) using Burrows–Wheeler Alignment. The remaining data by the removal of low-complexity reads were classified by simultaneously aligning to four Microbial Genome Databases, consisting of viruses, bacteria, fungi, and parasites. The classification reference databases were downloaded and optimized from public database, such as NCBI, EBI or Genbank. In the end, the multi-parameters of Species in Microbial Genome Databases were calculated and exported and professionals with microbiology and clinical background will interpret the result.

## Results

### Patients’ characteristics

Three members of an extended family of 6 persons developed *C. psittaci* pneumonia successively in Chongqing, China in December, 2020. Among them, two were women, one was man. Their median age was 56 (range 46–72) years (Table 1). All patients were positive for *C. psittaci* DNA fragments detected by mNGS, and systematic screening for other common pathogens for CAP, including COVID-19, H1N1 flu virus, parainfluenza virus, *Legionella pneumophila*, *Chlamydia pneumoniae*, *Mycoplasma pneumoniae* via serology, and blood culture, sputum culture, were all negative on admission to our hospital. All the patients had a definite exposure history, because they had all raised two new bought pet parrots in succession at home for 1 month, which had died 1 week before the admission. Three patients were in charge of taking care of these two parrots, including feeding and cleaning the feces, while the other 3 healthy family members only watched the parrots occasionally. Because they kept pet parrots due to the social isolation and restrictions on entertainment measures caused by the epidemic of COVID-19, it led to familial clustering in the meantime.

**Table 1.** Clinical characteristics of the psittacosis pneumonia cases.

Patient	1	2	3	Patients, *n* (%)	Median value (range)
*Demographics*					
Male/female	F	F	M	1/2	
Age, median(range, years)	72	40	46		56 (40–72)
History of contact with birds and poultry	Yes	Yes	Yes	3/3 (100.0)	
Underlying disease	No	No	Hepatitis B	1/3 (33.3)	
*Clinical manifestations*					
Fever > 38.5°C	Yes	Yes	Yes	3/3 (100.0)	
Weakness	Yes	Yes	Yes	3/3 (100.0)	
Poor appetite	Yes	No	Yes	2/3(66.7)	
Cough	Yes	Yes	No	2/3 (66.7)	
Chill	Yes	Yes	No	2/3 (66.7)	
Myalgia	No	Yes	No	1/3 (33.3)	
Date of admission	2020.12.22	2020.12.23	2020.12.28		
Days from the onset of the illness to admission	4	7	3		4.7 (3–7)
*Laboratory examination*					
WBC (normal 3.5–9.5 × 10^9^/L)	9.9	4.3	7.8		7.3 (4.3–9.9)
Percentage of neutrophils (normal 45–75%)	86.7%	58.8%	83.5%		76.3% (58.8–86.7%)
CRP(normal <10 mg/L)	187.2	56.8	155.0		133.0 (56.8–187.2)
PCT (normal 0.02–0.05 ng/ml)	0.64	0.09	0.64		0.45 (0.09–0.64)
CK (normal 38–174 U/L)	755	Normal	Normal		
LDH (normal 120–250 U/L)	335	370	257		
ALT (normal 7–40 U/L)	54	267	Normal		
AST (Normal 13–35 U/L)	91	108	Normal		
Hypokalaemia (Normal 3.5–5.2 mmol/L)	2.81	No	No		
*Imagings*					
Lesion began in the lower lobe of the lung	Yes	Yes	Yes		
Consolidation with air bronchograms	Yes	Yes	Yes		

Note: CK: creatine kinase, CRP: C-reactive protein, CT: computed tomography, LDH: lactate dehydrogenase, PCT: procalcitonin, WBC: white blood cell, ALT: alanine aminotransferase, AST: aspartate aminotransferase.

All patients had similar symptoms: recurrent fever higher than 38.5°C and weakness. Two patients had chill, cough and poor appetite, and one patient had myalgia at the onset of their illness. The median time from the onset of the illness to admission to our department was 4.7 (range 3–7) days (Table 1).

The first patient admitted to our hospital on 22 December 2020 was the 72-year-old female with the chief complaints of weakness for 4 days and fever for 8 h. Chest CT examination performed in our hospital on 22 December 2020 showed signs of consolidations with bronchograms which were compatible with pneumonia on the left lower lobe and the right upper lobe.

The second patient admitted to our hospital on 23 December 2020 was a 40-year-old female who was the elder daughter-in-law of the first patient, with the chief complaints of fever for 7 days and cough for 2 days. Chest CT examinations performed in another hospital on 21 December 2020 showed signs of infiltrates, reticular shadows and consolidations with bronchograms on the right lower lobe and the right upper lobe which were compatible with pneumonia.

The third patient admitted to our hospital on 28 December 2020 was the 46-year-old male who was the younger son of the first patient, with the chief complaints of weakness for 3 days and fever for 1 d. Chest CT examinations performed in our hospital on 28 December 2020 showed signs of consolidations with bronchograms on the right lower lobe which were compatible with pneumonia.

The signs on physical examination of the patients were rare and non-specific, only weakened respiratory sounds, and little moist rales on auscultation on the affected side were found in the first patient (the eldest patient).

### Laboratory examinations

**Figure 1.**
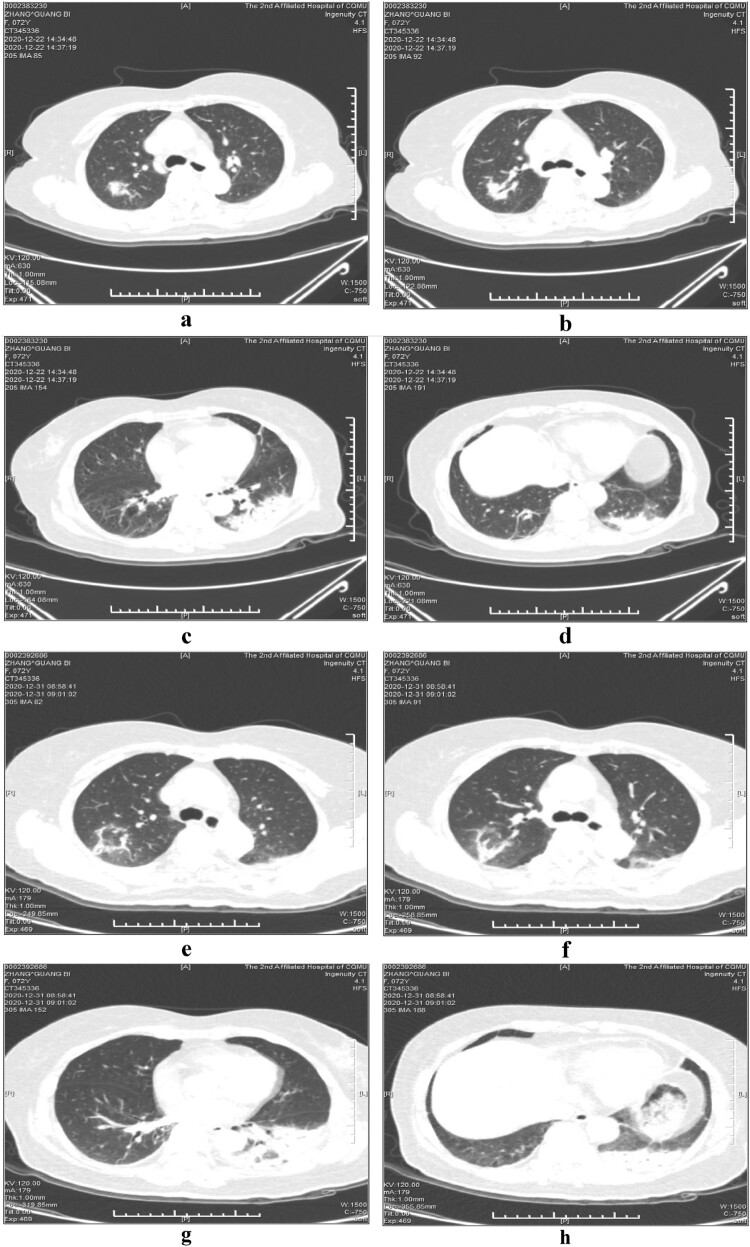
Serial chest computed tomography (CT) scans of the 72-year-old female with psittacosis pneumonia (the first patient). The initial CT scan (4 days after the onset) shows consolidations with bronchograms in the right upper lobe (a, b) and left lower lobe (c, d). The follow-up CT scan (13 days after the onset) shows the area of consolidation in the right upper lobe has decreased (e, f), while the consolidations in the left lower lobe only changed a little (g, h).

**Figure 2.**
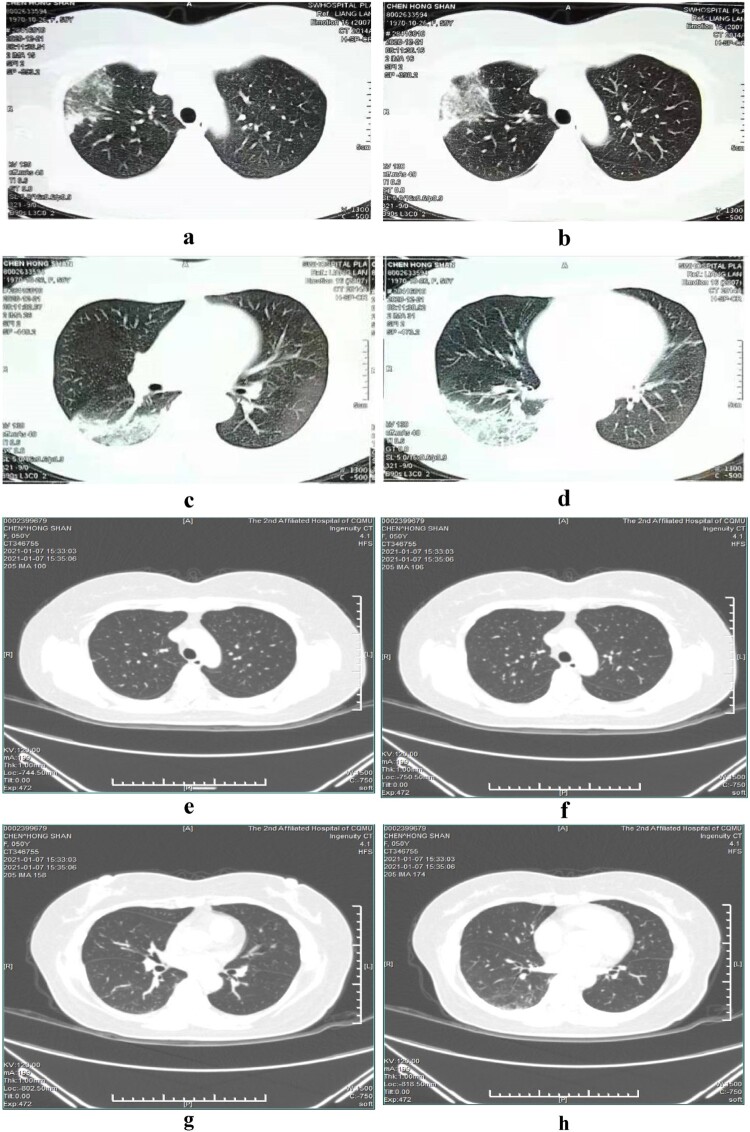
Serial chest computed tomography (CT) scans of the 40-year-old female with psittacosis pneumonia (the second patient). The initial CT scan (5 days after the onset) shows infiltrates, reticular shadows and consolidations with bronchograms in the right upper lobe (a, b) and right lower lobe (c, d). The follow-up CT scan (22 days after the onset) shows the area of infiltrates and consolidations in the right upper lobe (e, f) and right lower lobes (g, h) have both disappeared.

**Figure 3.**
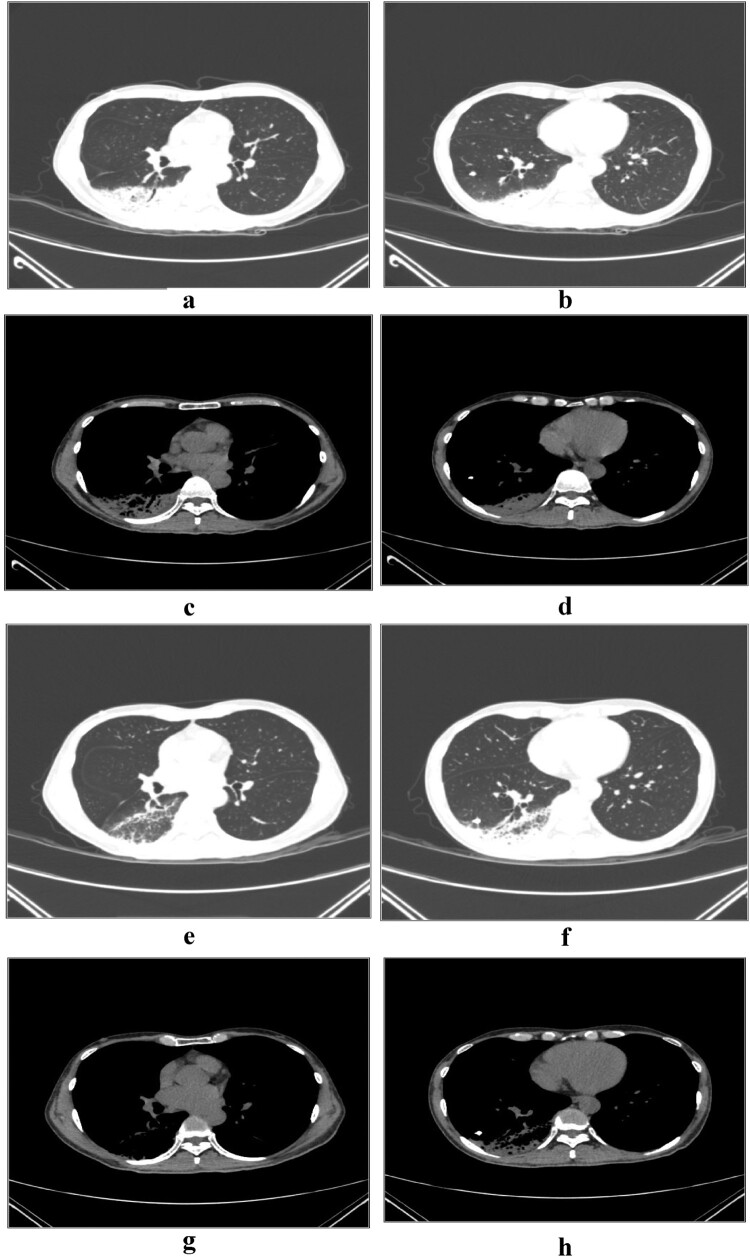
Serial chest computed tomography (CT) scans of the 46-year-old male with psittacosis pneumonia (the third patient). The initial CT scan (3 days after the onset) shows consolidations with bronchograms in the right lower lobe (a, b, c, d). The follow-up CT scan (13 days after the onset) shows the area of consolidation in the right lower lobe has decreased obviously (e, f, g, h).

On admission, the patients had a mean white blood cell count of 7.3 × 10^9^/L, percentage of neutrophils of 76.3%, C-reactive protein (CRP) level of 133 mg/L, and procalcitonin (PCT) level of 0.45 ng/mL. All the three patients had elevated lactate dehydrogenase levels, two patients had elevated alanine aminotransferase and aspartate aminotransferase levels, and one patient had elevated creatine kinase levels and hypokalaemia (Table 1). The inflammatory lesions were observed in the lower lobe of the lung in all the three patients, usually unilateral. The lobes were involved bilaterally in one patient (the eldest patient). Air-space consolidations, along with ground-glass opacities and reticular shadows, could be detected on CT scan. After clinical recovery, the inflammatory lesions decreased and disappeared gradually, with no residual fibrosis (Figures 1–3).

### mNGS results

**Figure 4.**
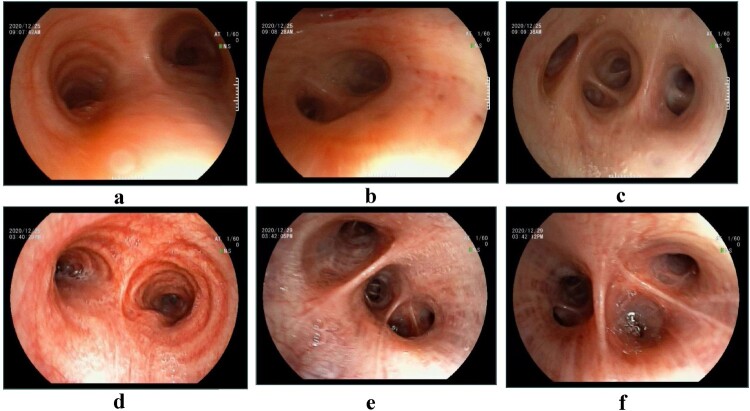
Bronchoscope imagings of patient 2 (a, b, c) and patient 3 (d, e, f). The typical bronchoscopy findings were hyperaemic tracheal and bronchial mucosa with oedema, with little thin white secretions in the segmental bronchi.

After admission to our hospital, two younger patients (patient 2 and patient3) underwent bronchoscopy, and bronchoalveolar lavage fluid (BALF) was collected for mNGS. The blood and sputum samples, while not BALF of the elderly patient, were simultaneously sent for mNGS due to the age and relatively poor condition. The typical bronchoscopy findings were hyperaemic tracheal and bronchial mucosa with oedema, with little thin white secretions in the segmental bronchi (Figure 4**)**.

**Figure 5.**
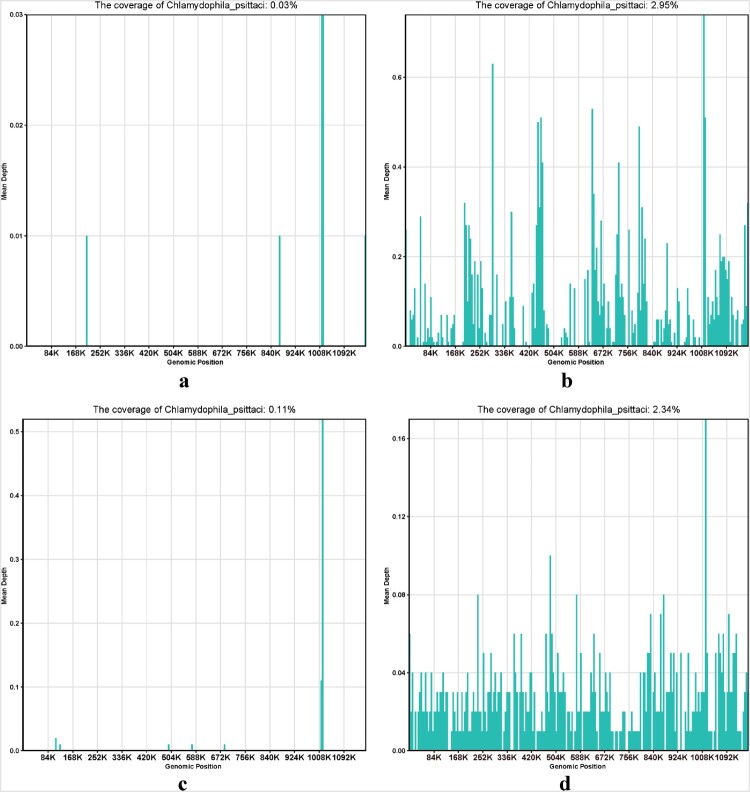
Metagenomic next generation sequencing results of patient 1, 2, 3. (a) Three specific *C. psittaci* sequences that covered 0.03% of the total *C. psittaci* genome were detected by mNGS in the blood sample of patient 1, (b) A total of 379 specific *C. psittaci* sequences that covered 2.95% of the total *C. psittaci* genome were detected by mNGS in the sputum sample of patient 1. (c) Two specific *C. psittaci* sequences that covered 0.11% of the total *C. psittaci* genome were detected by mNGS in the BALF sample of patient 2. (d) A total of 779 specific *C. psittaci* sequences that covered 2.34% of the total *C. psittaci* genome were detected by mNGS in the BALF sample of patient 3.

Three specific *C. psittaci* sequences that covered 0.03% of the total *C. psittaci* genome were detected by mNGS in the blood sample of the first patient, and 379 specific *C. psittaci* sequences that covered 2.95% of the total *C. psittaci* genome were detected by mNGS in the sputum sample of the first patient. Two specific *C. psittaci* sequences that covered 0.11% of the total *C. psittaci* genome were detected by mNGS in the BALF sample of the second patient. A total of 779 specific *C. psittaci* sequences that covered 2.34% of the total *C. psittaci* genome were detected by mNGS in the BALF sample of the third patient (Table 2 and Figure 5).

**Table 2.** mNGS results of the psittacosis pneumonia cases.

Patient	1	2	3
Detected pathogens and specific reads (*n*)	Sputum: *Chlamydia psittaci (379), Lactobacillus gasseri (10,941), Lactobacillus salivarius (4,493), Enterococcus faecalis (1,296), Haemophilus parainfluenzae (203), Tropheryma whipplei (191), Candida albicans (2,101)* Blood: *Chlamydia psittaci (3)*	BALF: *Chlamydia psittaci (2)*, *Haemophilus parainfluenzae (97)*	BALF: *Chlamydia psittaci (563)*

Note: BALF: bronchoalveolar lavage fluid.

## Treatments

Prior to admission, the second patient went to another hospital for help. The initial treatment was antipyretic (paracetamol) to control the symptoms, but it didn’t work. Then clinician in that hospital added a cephalosporin (cefdinir). The first and third patients didn’t go to hospital, and took some antipyretics (paracetamol) by themselves. However, the clinical condition didn’t improve but gradually deteriorated.

Patients were given empirical antibiotic therapy with β-lactam/β-lactamase inhibitor combinations and quinolones on admission according to the CAP management guideline [21]. The mNGS took 48–72 h from the receipt of the samples to the reporting of the results. When *C. psittaci* infection was confirmed, the antibiotic was changed to minocycline [22]. Minocycline was administered for at least 2 weeks, according to recommendations [22]**.**

## Outcomes

After minocycline therapy was initiated, patients’ fever generally disappeared within 3 days, and their cough and general conditions, including weakness and poor appetite, gradually improved. The percentage of neutrophils reduced to normal. Procalcitonin and C-reactive protein dropped sharply to near normal (Table 3). Consolidations and infiltrates on CT imagings absorbed obviously 13 days after the onset in patient 3, disappeared 22 days after the onset in patient 2, and decreased slowly 10 days after the onset in patient 1 (the eldest patient). But the clinic conditions of patient 1 had alleviated evidently at that time.

**Table 3.** Values of infection indexes before and after minocycline therapy for the psittacosis pneumonia cases.

Patient	1	2	3	1	2	3
Laboratory examination	Before minocycline therapy			After minocycline therapy		
WBC (normal 3.5–9.5 × 10^9^/L)	9.9	4.3	7.8	6.9	5.1	4.5
Percentage of neutrophils (normal 45–75%)	86.7%	58.8%	83.5%	64.7%	66.7%	64.9%
CRP (normal <10 mg/L)	187.2	56.8	155.0	27.8	10.8	6.9
PCT (normal 0.02–0.05 ng/ml)	0.64	0.09	0.64	0.05	0.05	0.02

Note: **WBC:** white blood cell, **CRP**: C-reactive protein, **PCT**: procalcitonin.

## Discussion

Psittacosis is a common disease in birds caused by the infection of *Chlamydia psittaci* [23, 24]. However, psittacosis in human beings is considered uncommon, which is usually sporadic [3, 4]. There were a few reports of human psittacosis outbreaks in European countries, USA, Australia, Japan etc. [24–43]. There were even fewer reports of family outbreaks of psittacosis [8, 10, 12, 13, 44–47]. Hitherto, there has been no report of an outbreak of human psittacosis from China, and nor family outbreak.

In this paper, we describe the first reported family outbreak of human psittacosis in China, to our knowledge. As to the reasons for relatively fewer reports of human psittacosis in China, especially outbreaks, except a lower rate of pathogen carriers among Asian parrots than among European parrots [48], the limitations of detecting methodology may be a major one. The current existing testing methods for *C. psittaci* can be divided into various serology, namely IF, CFT, ELISA/EIA, and IPA tests, culture, and molecular detections. Culture has high specificity and is often used to confirm the diagnosis, but is generally used as a supplement to other tests and requires a high-level biosafety laboratory. Hence culture cannot be routinely performed in most diagnostic laboratories and hospitals. Serological tests were used in most reports [8, 13, 30, 31, 39, 41, 49]. In many articles, problems occurred with the unavailability of convalescent serum samples required for confirmation of cases or the failure of the second sample to show seroconversion, which makes interpretation and conclusions difficult. In the meantime, due to the cross-reactivity with other Chlamydial species, serological tests of a serum sample taken at one single moment are insufficient for confirmation [50]. Molecular detection methods are used in recent 20 years. PCR is used most commonly [35, 37, 38, 41]. However, PCR testing for *C. psittaci* is not available in most hospitals in China, including many tertiary hospitals. PCR is a highly specific targeted test, but it is usually performed only when clinicians suspect the infection of *C. psittaci*, which is difficult to diagnose clinically.

In recent years, mNGS, another molecular detection method came into our view. The advantage of mNGS is that it has a wide detection range and does not need to specify the suspected causative microorganism priorly, and can be used in the diagnosis of encephalitis, meningitis, and lower respiratory tract infections [51, 52]. In our hospital, mNGS results can be obtained in 48–72 h, but routine sputum culture takes 5–7 days, and many cultures are negative. The ability of mNGS to obtain timely and precise microbial diagnoses of infections is a key advantage. When treating patients with pneumonia of unknown pathogen and severe pneumonia, physicians need to identify the causative pathogen as early as possible, and to make the accurate diagnosis to provide targeted treatment. Because of atypical clinical features and the diagnostic challenges, psittacosis is often misdiagnosed. It is worthwhile to use mNGS in patients with pneumonia to minimize the time to diagnosis of psittacosis and the course of the disease.

In our study, it was lucky for these three patients in this family outbreak. After screening other common pathogens for CAP including COVID-19, H1N1 flu virus, parainfluenza virus, *Legionella pneumophila*, *Chlamydia pneumoniae*, *Mycoplasma pneumoniae* via serology, and other pathogen through blood culture, sputum culture on admission which were negative, and giving empiric treatment with antibiotics for CAP which didn’t work, they were given mNGS of blood and bronchoalveolar lavage fluid to detect the pathogen, especially after a family cluster trend was observed. The results of mNGS returned to us within 48 h and identified *C. psittaci* in all the three patients. After we got these results, we retrospected the contact history of birds and poultry, a definite exposure history of two sick pet parrots, which had died 1 week before the admission, was traced. The use of mNGS can improve accuracy and reduce the delay in the diagnosis of psittacosis especially during the outbreak, which permits a timely study of the spread of the disease and shortens the course of the disease control.

With the use of mNGS, there is a growing number of human psittacosis identified in clinic practice in China, especially among fever of unknown origin and atypical pneumonia caused by rare pathogens. Due to the non-specific symptoms and imaging manifestations, psittacosis is easily underdiagnosed and misdiagnosed. This may be another reason for the low report rate in China.

The social isolation and restrictions on recreational measures due to COVID-19 may lead to increased demand for pets. Among them, pet birds especially parrots, will increase the risk of psittacosis. At the same time, the epidemic of COVID-19 decreases unnecessary travel which leads to relatively increase of familial aggregation and the possibility of family outbreak of psittacosis.

Our three cases of psittacosis pneumonia, diagnosed with the help of mNGS, have similar clinical features and characteristics with previously reported cases [16, 53]. Previous reports of psittacosis have included fever, cough, chills, myalgia, and weakness as general symptoms, and most reported cases have been of mild-to-middle severity [22, 54]. In our three cases, fever and weakness were prominent features in all patients throughout the whole course of disease, which were analogous with flu-like general symptoms. Two patients had the respiratory symptom of cough, and the digestive symptom of poor appetite as the disease progressed. One patient had the symptom of myalgia. Hepatic involvement, mainly manifesting as mild elevations of alanine aminotransferase and aspartate aminotransferase, was detected in two patients on admission, which was considered a rare complication of psittacosis [13]. Interestingly, the patient with the underlying disease of hepatitis B didn’t develop hepatic function damage, while the other two patients without any underlying disease developed. After treated with minocycline, the liver function became normal gradually. This indicates the hepatic function damage caused by psittacosis may have individual variation, not depending on whether there is underlying hepatopathy.

The laboratory data of patients in our paper showed normal or slightly elevated leucocytes, neutrophils, and PCT, along with high CRP levels, which are consistent with those usually showed in psittacosis. Knittler and Sachs [55] reported that *C. psittaci* has higher pathogenicity and faster reproduction rate than other *Chlamydiales* species, so *C. psittaci* causes more severe inflammatory reactions. Inflammatory lesions representing, as consolidation with bronchograms and ground-glass opacity close to the pleura on CT, were mainly in the lower lobe of the lung in all the three patients, and also in the upper lobes in two patients, which were more extensive on radiography than would be expected from clinical signs. In the meantime, the manifestations under bronchoscope, including congestion and oedema of bronchial mucosa, and the amount of secretions in the airway, were lighter than would be expected depending on the range of consolidations, which are different from CAP with similar CT imagings caused by bacteria. The characteristic appearances of psittacosis under bronchoscope have not been reported previously.

As *C. psittaci* belongs to the family *Chlamydiaceae* in the order *Chlamydiales* [17], tetracyclines, macrolides, and quinolones, which can interfere with DNA and protein synthesis, can be used as antibiotic therapy [56]. Tetracyclines, including tetracycline hydrochloride and doxycycline, are generally considered to be the first-line treatment for psittacosis [57]. Minocycline, a second-generation tetracycline, also cures *C. psittaci* infections, with a minimum inhibitory concentration of 0.03–0.06 mg/L and a minimum bactericidal concentration of 0.06–0.25 mg/L in vitro [58]. Because of its higher efficacy and lower incidence of side effects, our patients were treated with minocycline instead of tetracycline or doxycycline. And all patients responded to minocycline quickly. Fever disappeared first followed by cough, poor appetite and weakness. The inflammatory lesions on CT also absorbed obviously 13 days later in patient 3, and absorbed completely 22 days later in patient 1. However, the lesions only absorbed lightly 10 days later in patient 1 who was also the eldest, although at that time the patient almost had no clinical symptoms and signs. This indicates that imaging recovery is later than clinical recovery, especially in elder patients. Hence it is necessary to trace the imaging changes continuously to know the degree of recovery, even though the clinical manifestations have disappeared.

In outbreaks of psittacosis, there is often a considerable delay between the onset of symptoms in patients and the laboratory diagnostic test, especially when the history is not taken carefully and the bird contact is unknown, standard diagnosis in patients with pneumonia usually does not include tests for *C. psittaci* infection. As a result, many psittacosis index patients are discovered late and often in hospital. mNGS can quickly and objectively detect more and more rare pathogenic microorganisms in clinical specimens, without the need for specific amplification, and has an advantage in the diagnosis of rare pathogenic bacteria in difficult cases. The use of mNGS can improve the accuracy and reduce the delay in the diagnosis of psittacosis especially during the outbreak, which permits a timely study of the spread of the disease and shortens the course of the disease control. It may be a better testing method for *C. psittaci* than culture, PCR and various serological methods, particularly in outbreaks of psittacosis. However, the cost of mNGS is relatively high in clinic, so it can not completely replace the current conventional identification methods. And limitations to the use of mNGS technology exist, despite its widespread use. There is no authoritative guide to the interpretation of the mNGS report. Detection of a broad spectrum of pathogens by mNGS has blunted the diagnosis of pathogenicity resulting in the inability to distinguish between background, colonization and microbial infection, and pollution [59]. Hence, the interpretation of mNGS report must combine with clinic. Clinical comprehensive thinking is still the core of the diagnosis and treatment of infectious diseases, and mNGS just provides a new approach to this core.

The main limitation of this study is that it included only three cases of psittacosis pneumonia. This relatively small sample size is insufficient to investigate all the relevant features of psittacosis pneumonia. The study was a retrospective study and we did not use CFT or MIF to confirm the diagnosis. Given the poor response of CAP to empirical antibiotics, mNGS can shorten the time to diagnosis and enable the initiation of targeted antibiotic therapy earlier.
